# Implementing a lifestyle toolkit in individuals with cardiometabolic multimorbidity: study protocol for a cluster randomised, type III effectiveness–implementation trial (SHRADDHA-CMM)

**DOI:** 10.1186/s13063-026-09793-2

**Published:** 2026-06-02

**Authors:** Aswathy Sreedevi, Chandrasekhar Janakiram, Navami Sasidharan, Vishnu B. Menon, Divya CS, Veena G. Nair, Aravind MS, Bipin Gopal, Mathews Numpeli, Aarati Krishnan, Jaya Rajeev, Malathi Mini, Parvathy Balachandran, Georg Gutjahr, Philip Morisky, Tanveer Rehman, Sanghamitra Pati, Jaideep C. Menon

**Affiliations:** 1 Amrita Institute of Medical Sciences, Amrita Vishwa Vidyapeetham, Kochi, Kerala India; 2 Directorate of Health Services, Government of Kerala, Thiruvananthapuram, Kerala India; 3 District Medical Office (H), Government of Kerala, Ernakulam, India; 4 Adherence LLC, Long Beach, USA; 5 ICMR-Regional Medical Research Centre, Bhubaneswar, India; 6 ICMR Headquarters, New Delhi, India

**Keywords:** Cardiovascular disease, Cardiometabolic multimorbidity (CMM), ENDIRA, Cluster randomised controlled trial (c-RCT), SHRADDHA lifestyle toolkit

## Abstract

**Abstract:** Lifestyle modification, including dietary interventions, physical activity, and stress management, has proven crucial in the non-pharmacological management of cardiometabolic diseases. However, the implementation of these interventions in the context of cardiometabolic multimorbidity (CMM) within the unique demographic and cultural milieu of India is inadequately explored, despite Indians exhibiting distinct susceptibility to cardiovascular diseases. This community-based study aims to address existing knowledge gaps by implementing a culturally tailored lifestyle toolkit based on the WHO-HEARTS framework among individuals with CMM and exploring the intergenerational preventive dimension. The study adopts a two-arm non-blinded cluster randomised controlled trial (c-RCT) design to include adults diagnosed with CMM, defined by the presence of two or more of diabetes mellitus, hypertension, stroke, or coronary heart disease, aged 18 years and above, providing informed consent, and agreeing to follow-up. The trial spans the ‘Epidemiology of Non-communicable Diseases In Rural Areas’ (ENDIRA) cohort in Ernakulam, Kerala. The intervention, ‘SHRADDHA lifestyle toolkit’, integrates guidance on diet, physical activity, stress management, tobacco and alcohol cessation, and drug adherence. It leverages frontline health workers and a digital health platform for implementation. Measurements involve use of point-of-care devices and digital tools. The study duration is 24 months, with a sample size of 2000 participants distributed across 18 clusters. Statistical analysis includes repeated-measures ANOVA and multilevel models, employing an intention-to-treat approach. The study’s novelty lies in its holistic approach, addressing various facets of CMM comprehensively.

**Trial registration: **The trial has been registered with the Clinical Trial Registry of India (CTRI) Reference Number—CTRI/2025/08/092529. Registered on August 6, 2025

## Administrative information

Note: The numbers in curly brackets in this protocol refer to SPIRIT checklist item numbers along with spirit guidance. The order of the items has been modified to group similar items.

**Table:** 

Title and structured summary	
Title stating the trial design, population, and interventions, with identification as a protocol {1a}	Implementing a lifestyle toolkit in individuals with cardiometabolic multimorbidity: protocol for a type III effectiveness–implementation trial (SHRADDHA-CMM)
Trial registration	
Name of trial registry, identifying number (with URL), and date of registration. If not yet registered, name of intended registry {2a}	Registered in Clinical Trials Registry—India: CTRI/2025/08/092529, dated 06/08/2025 https://ctri.nic.in/Clinicaltrials/rmaindet.php?trialid=99043&EncHid=16423.46502&modid=1&compid=19
Structured summary of trial design and methods, including items from the World Health Organization Trial Registration Data Set {2b}	Abstract contains background, design (two-arm non-blinded cluster RCT, type III implementation–effectiveness), setting (ENDIRA cohort, Ernakulam, Kerala), sample size (2000 across 18 clusters), intervention summary, outcomes, and main timepoints (baseline, 6, 12 months)
Protocol version	
Version date and identifier {3}	Version 4; date: 26 September 2025
Funding	
Sources of funding and other support (e.g. supply of drugs) {4}	This research study is supported by the Alliance for Health Policy and Systems Research
Roles and responsibilities	
Names, affiliations, and roles of protocol contributors {5a}	Aswathy Sreedevi, Professor and Head, Department of Community Medicine, Amrita Institute of Medical Sciences, Amrita Vishwa Vidyapeetham, Kochi Chandrasekhar Janakiram, Professor and Head, Department of Public Health Dentistry, Amrita School of Dentistry, Amrita Vishwa Vidyapeetham, Kochi, India Navami Sasidharan, Assistant Professor, Department of Community Medicine, Amrita Institute of Medical Sciences, Amrita Vishwa Vidyapeetham, Kochi Vishnu B Menon, Assistant Professor, Department of Community Medicine, Amrita Institute of Medical Sciences, Amrita Vishwa Vidyapeetham, Kochi Divya C S, Project Coordinator, CMM WHO Project, Amrita Institute of Medical Sciences, Amrita Vishwa Vidyapeetham, Kochi Veena G Nair, Project Technical Staff, Amrita Institute of Medical Sciences, Amrita Vishwa Vidyapeetham, Kochi Aravind M S, Junior Consultant, Amrita Institute of Medical Sciences, Amrita Vishwa Vidyapeetham, Kochi Bipin Gopal, State Nodal Officer NCD, DHS, Government of Kerala, Thiruvananthapuram, India Mathews Numpeli, State Nodal Officer (Palliative Care) and Assistant Surgeon, FHC Kalloorkad, DHS, Government of Kerala, India Aarati Krishnan, District Nodal Officer (Non Communicable Diseases), DMO (H), Ernakulam, Kerala, India Jaya Rajeev, Assistant Surgeon, CHC Kalady, DHS, Government of Kerala, India Malathi Mini, Assistant Professor, Department of Public Health, Amrita Institute of Medical Sciences, Amrita Vishwa Vidyapeetham, Kochi Parvathy Balachandran, Assistant Professor, Department of Public Health Dentistry, Amrita School of Dentistry, Amrita Vishwa Vidyapeetham, Kochi, India Georg Gutjahr, Assistant Professor, Biostatistics, Health Sciences Research, Amrita Institute of Medical Sciences, Amrita Vishwa Vidyapeetham, Kochi Philip Morisky, Adherence LLC, Long Beach, USA Tanveer Rehman, Scientist C, ICMR-Regional Medical Research Centre, Bhubaneswar, India Sanghamitra Pati, Additional Director General, ICMR Headquarters, New Delhi, India Jaideep C Menon*, Professor, Department of Adult Cardiology & Public Health, Amrita Institute of Medical Sciences, Amrita Vishwa Vidyapeetham, Kochi
Name and contact information for the trial sponsor {5b}	WHO-Alliance for Health Policy and Systems Research
Role of trial sponsor and funders in design, conduct, analysis, and reporting of trial; including any authority over these activities {5c}	Study sponsor does not have any role in the collection, management, analysis, and interpretation of data
Composition, roles, and responsibilities of the coordinating site, steering committee, endpoint adjudication committee, data management team, and other individuals or groups overseeing the trial, if applicable {5d}	Not applicable
Protocol and statistical analysis plan	
Where the trial protocol and statistical analysis plan can be accessed	Can be accessed in the CTRI website
Data sharing	
Where and how the individual deidentified participant data (including data dictionary), statistical code, and any other materials will be accessible {29}	Deidentified participant data will be shared on an individual request basis
Conflicts of interest	
Financial and other conflicts of interest for principal investigators and steering committee members {7b}	The authors declare that there are no competing interests
Dissemination policy	
Plans to communicate trial results to participants, healthcare professionals, the public, and other relevant groups (e.g. reporting in trial registry, plain language summary, publication) {31}	The results will be shared through peer-reviewed publication, the public and research community, and policy makers

## Introduction

### Background and rationale {6a}

Multimorbidity, defined as the coexistence of two or more chronic conditions in an individual, has emerged as a significant global health challenge, affecting nearly one-third of the global population [1, 2]. Its burden is particularly pronounced in low- and middle-income countries (LMICs), where rapid demographic and epidemiological transitions are driving rising prevalence [3]. In India, estimates of multimorbidity vary widely, ranging from 8.9 to 57% across studies [4]. Despite its ubiquity, research on multimorbidity in LMICs particularly in primary care and community settings remains limited and fragmented [5].

Cardiometabolic multimorbidity (CMM), a subset of multimorbidity, is especially important given its high prevalence, clinical complexity, and significant impact on mortality and disability [6]. CMM refers to the coexistence of two or more cardiometabolic conditions, including hypertension, diabetes, coronary heart disease (CHD), and stroke. Globally, cardiovascular diseases (CVDs)—the principal component of CMM—constitute a major health burden, with CHD and stroke ranking among the leading causes of mortality [7]. The situation in India is particularly concerning: CVD mortality rates (282 per 100,000) exceed the global average (233 per 100,000), accounting for more than one-quarter of total deaths and 14% of disability-adjusted life years (DALYs) lost [8].

Lifestyle modification—encompassing diet, physical activity, and stress management—has long been recognised as a cornerstone of non-pharmacological strategies for cardiometabolic risk reduction [9, 10]. Dietary interventions play a critical role in glycaemic control, with evidence demonstrating significant reductions in HbA1c through low-carbohydrate diets [11]. Similarly, the Dietary Approaches to Stop Hypertension (DASH) diet has consistently lowered blood pressure in diverse populations [12]. Nutritional interventions also support weight management by promoting energy balance, thereby reducing BMI and associated risks [13]. Beyond diet, cessation of tobacco and alcohol use exerts additional benefits; smoking cessation is associated with improvements in HbA1c and reductions in blood pressure [14, 15]. Stress management practices further contribute to better glycaemic control and lower blood pressure [16]. Physical activity is another key determinant, with robust evidence linking regular exercise to improved glycaemic control, lower blood pressure, and reduced cardiovascular risk. In addition, adherence to prescribed medications remains critical for secondary prevention among individuals with CHD or stroke and for maintaining blood pressure and glucose within recommended targets [17].

Although these interventions are well supported by global evidence, their effectiveness in the context of CMM, particularly within the diverse demographic, cultural, and health system contexts of India remains poorly studied [18]. Notably, Asian Indians exhibit increased susceptibility to cardiometabolic disease, often experiencing onset at younger ages compared to Western populations [19]. The burden is not uniform across India; for example, Kerala reports the highest prevalence of CVD of all Indian states [20]. Moreover, Indians frequently present with more severe manifestations, driven by genetic predispositions, environmental exposures, and lifestyle factors. This unique interplay underscores the need for tailored preventive and management strategies.

Several interventions worldwide have demonstrated the effectiveness of lifestyle modification in improving cardiovascular outcomes. The WHO-HEARTS technical package has been implemented in multiple LMICs, including India, showing feasibility in strengthening hypertension and CVD management through standardised protocols and primary care integration [21–23]. Similarly, trials in South Asia have shown that dietary counselling, tobacco cessation support, and promotion of physical activity can lead to improvements in blood pressure, glycaemic control, and body weight [24, 25].

Community health worker (CHW)–led interventions have been effective in bridging gaps in healthcare delivery, particularly for hypertension and diabetes control in underserved populations. For example, the HOPE-4 trial demonstrated that CHW-delivered lifestyle interventions, combined with simple drug regimens, reduced blood pressure and cardiovascular risk in LMICs [26]. In India, CHWs have successfully delivered structured counselling and follow-up for diabetes and hypertension management [27, 28].

Digital and mobile health (mHealth) platforms have further enhanced the delivery of lifestyle interventions, providing real-time feedback, reminders, and behaviour-change communication. Evidence from India and other LMICs shows that mHealth tools improve medication adherence, support tobacco cessation, and enhance dietary and physical activity practices [29–31].

Despite this promising evidence, important gaps remain. Most interventions have targeted single conditions such as hypertension or diabetes, rather than addressing the complex clustering of conditions characteristic of cardiometabolic multimorbidity (CMM). Few studies have evaluated integrated, multi-component toolkits combining diet, physical activity, stress management, substance cessation, and medication adherence in a unified approach tailored for CMM. While CHWs and mHealth platforms have been studied separately, evidence on their combined use for delivering structured, culturally tailored interventions in multimorbidity is limited. Importantly, the intergenerational dimension of cardiometabolic risk prevention has been largely overlooked, despite evidence of familial clustering of cardiovascular risk. Finally, very few interventions in India have explicitly adapted the WHO-HEARTS framework into a community-delivered lifestyle package that is regionally contextualised.

The Shraddha lifestyle toolkit is a culturally tailored adaptation of the WHO-HEARTS framework for South India. It integrates community health worker–led counselling with mHealth support to promote diet, physical activity, stress reduction, tobacco and alcohol cessation, and medication adherence. The toolkit will evaluate both clinical (HbA1c, blood pressure, BMI, adherence) and implementation outcomes (acceptability, adoption, cost). Uniquely, it also addresses the intergenerational dimension of cardiometabolic risk.

In addition, there is growing recognition of the intergenerational transmission of cardiometabolic risk. Evidence suggests that early-life exposures and parental health status can shape the cardiovascular health trajectories of offspring, highlighting the need for preventive strategies that extend beyond individual patients to families and future generations [32].

Against this background, our study seeks to address critical knowledge gaps by evaluating the effectiveness of a culturally tailored lifestyle intervention, adapted from the WHO-HEARTS framework, in improving outcomes among individuals with CMM in India [21]. Furthermore, by exploring the intergenerational dimension of CMM prevention, this study aims to generate insights that could inform innovative, family-centric strategies to mitigate the long-term burden of cardiometabolic disease.

We aim to bridge existing knowledge gaps by implementing a culturally tailored lifestyle toolkit based on the WHO-HEARTS framework in improving health outcomes among individuals with CMM in India [21]. We also aim to study the intergenerational preventive dimension of CMM, an aspect that has so far received limited attention.

### Objectives {7}

- To identify the barriers and facilitators to implementing a lifestyle toolkit delivery model in individuals with cardiometabolic multimorbidity factoring for current lifestyle practices in managing cardiovascular disease (CVD).
- To co-develop and implement a context-specific lifestyle toolkit model tailored to the needs of the target population and support its iterative optimisation.
- To evaluate the optimised lifestyle toolkit model for acceptability, adoption, fidelity, coverage, and cost for implementation in a community setting.
- To evaluate the impact of the Shraddha lifestyle toolkit in improving glycaemia control (glycated haemoglobin), systolic and diastolic blood pressure, body mass index (BMI), and adherence to prescribed medications among individuals with cardiometabolic multimorbidity (CMM).

#### Research question

How can a co-developed, context-specific lifestyle toolkit achieve high coverage and equitable adoption of lifestyle practices for managing CVD among individuals with CMM in a community setting, using the WHO-HEARTS framework?

## Methodology

### Trial design {8}

Type III implementation–effectiveness using a two-arm non-blinded cluster randomised controlled trial (c-RCT) design.

### Trial setting {9}

The study is to be carried out in the ENDIRA cohort of Ernakulam, Kerala [22, 23]. The ENDIRA (‘Epidemiology of Non-communicable Diseases In Rural Areas’) cohort (*n* = 114,064) includes all individuals from five panchayats (villages) in Ernakulam district, Kerala. Four of the five panchayats have family health centres (FHCs), and one community health centre (CHC), which are further divided into 18 sub-centres and 75 wards having 2064 individuals with documented cardiovascular diseases. Among these individuals, 37.3% were diabetic, 53.5% hypertensive, 30.5% were dyslipidaemic, 16.8% were tobacco users, and 23.2% were alcohol users [24]. Leveraging previous research initiatives in this region ensures the seamless integration of the current study and enhances its generalisability to a broader context.

#### Rationale for selection

Leverages identified individuals with CMM from the ENDIRA cohort where there are high prevalence rates of cardiovascular diseases, aligning with the study’s focus on individuals diagnosed with diabetes, hypertension, stroke, or heart disease. Additionally, it builds on a WHO-Alliance–funded study which aimed at improving adherence to drugs for CVD in the same cohort.

### Sample size {14}

The sample size was determined using computer-generated randomisation at the cluster level across 18 sub-centres (9 intervention and 9 control).

As this is a type III implementation study, the primary focus will be on evaluating implementation outcomes. In addition, effectiveness will also be assessed based on the following sample size estimation.

To detect a minimum clinically meaningful difference of 0.5% in HbA1c between the two groups, with 80% power, a two-sided alpha of 0.05, and assuming a standard deviation of 1.5% [33], the required sample size under individual randomisation was calculated to be 142 participants per arm.

Given the cluster design, with a planned cluster size of 90 participants and an intracluster correlation coefficient (ICC) assumed to be 0.05, the design effect was calculated as 5.45 using the formula DE = 1 + (m − 1) × ICC. After adjusting for clustering, the required sample size was 774 participants per arm.

The trial will include nine clusters per arm, with 90 participants per cluster (810 participants per arm), which exceeds the minimum required sample size. The fixed cluster size and number of clusters are determined by the structure of the ENDIRA cohort, within which this follow-up study is embedded.

### Strategies for achieving adequate participant enrolment to reach target sample size {15}

To achieve the target sample size, the study will leverage community health workers (ASHAs) to conduct household-based recruitment, ensuring inclusive enrolment of all eligible participants within each cluster from a community-benefit perspective.

### Randomisation sequence generation {16a}

The study will employ simple randomisation at the cluster level and will randomise 18 sub-centres into intervention and control arms.

### Mechanism used to implement the random allocation sequence {16b}

Since randomisation is at cluster level, allocation will be revealed at cluster assignment.

### Whether the personnel who will enrol and those who will assign participants to the interventions will have access to the random allocation sequence {16c}

The allocation sequence will be generated by the statistician; frontline health workers will enrol participants at household level using questionnaires on a tablet PC; randomisation is at cluster (sub-centre) level and interventions delivered at sub-centre by Janakeeya Arogya Kendra (JAK) staff which includes junior health inspectors (JHI), mid-level service provider (MLSP), and junior public health nurses (JPHN).

### Blinding {24}

#### Who will be blinded after assignment to interventions {17a}

The trial is non-blinded. Participants and intervention staff are aware of allocation. Only data analysts will be blinded.

#### If blinded, how blinding will be achieved and description of the similarity of interventions

Participants in the control arm continue with the usual standard of care, while the lifestyle toolkit is implemented in the intervention arm, with each sub-centre (JAK centre) serving as a cluster. Each of the staff including the implementors at the sub-centres is aware of the intervention and in being in the intervention arm, the blinding is only for the data analyst, who is a statistician in this study.

#### If blinded, circumstances under which unblinding is permissible, and procedure for revealing a participant’s allocated intervention during the trial {17b}

Only the data analysts will be blinded. The data analyst will be unblinded if there are any outlier biochemical values which requires immediate action so that the patient can be intimated.

### Eligibility criteria for participants {10}

Inclusion criteria: individuals diagnosed with CMM (defined as the presence of two or more of the following conditions: diabetes mellitus, hypertension, stroke, or coronary heart disease); stroke would include a documented ischaemic or haemorrhagic cerebrovascular accident as diagnosed from symptoms or imaging studies; coronary heart disease as defined by individuals with a history of an acute coronary syndrome, or diagnosed coronary artery disease defined as 50% stenosis of one or more of the epicardial coronaries on CT or conventional coronary angiography; or wall motion abnormalities (WMA) on echocardiography or nuclear imaging suggestive of an old myocardial infarction; age ≥ 18 years, providing informed consent for participation and agreeing to follow-up.

Exclusion criteria: individuals with cognitive impairment who are unable to carry out instructions; bedridden individuals; individuals with other significant confounding health conditions additionally like malignancy or chronic airway disease; individuals ≥75 years of age; individuals with a terminal medical condition; pregnant or lactating mothers.

### Participant timeline {13}

**Table:** 

Timepoint	Trial period				
	Enrolment		Post-randomisation		Close-out
	(−ti to 0)	0	t1 (6 mo)	t2 (12 mo)	tx
Enrolment					
Eligibility screen	X				
Informed consent	X				
Baseline demographics	X				
Cluster randomisation (sub-centres: arm A/arm B)		X			
Intervention/comparator					
Shraddha lifestyle toolkit (arm A)		X			X
Standard of care (arm B)		X	X	X	X
Assessments					
Clinical measures (BP, weight, BMI)	X		X	X	X
Laboratory (HbA1c)	X		X	X	X
Diet (FFQ/calorie tracking)	X		X	X	X
Physical activity (GPAQ)	X		X	X	X
Substance use (WHO-ASSIST)	X		X	X	X
Medication adherence (MMAS-8)	X		X	X	X
Mental stress (PSS-10)	X		X	X	X
Quality of life (EQ-5D-L3)	X		X	X	X
Implementation outcomes (acceptability, adoption, appropriateness, fidelity, cost effectiveness)			X	X	X

Baseline = 0; t1 = 6 months (midline); t2 = 12 months (endline). The arrow indicates continuous delivery of the Shraddha lifestyle toolkit (mHealth messages, JAK visits, yoga sessions) from allocation to endline. Protocol v4 (26 September 2025); IEC ASM-AIMS ECASM-AIMS-2024-001; CTRI/2025/08/092529

## Trial status

Protocol version: v4, dated 26 September 2025.

Ethical approval: IEC ASM-AIMS ECASM-AIMS-2024-001.

Trial registration: Clinical Trial Registry of India (CTRI/2025/08/092529 registered on 06/08/25).

The training for baseline survey is completed, and enrolment will be initiated in the third week of February. The inclusion of the last patient is estimated to be in April 2026. Study close is expected by November 2027.

Each participant will be followed up for 12 months, corresponding to the intervention duration, and the total study duration is 24 months, including formative, implementation, and analysis phases. Primary outcome assessments will be conducted at baseline (0 months), 6 months (midline), and 12 months (endline).

## Implementation aspects

### Intervention and comparator {11}

The intervention consists of a ‘lifestyle toolkit’, called ‘Shraddha’ (means ‘care’ in the local language, Malayalam), that includes guidance on diet and nutrition, tobacco and alcohol cessation, stress management, physical activity, and drug adherence. This toolkit is developed based on the WHO-HEARTS framework with some regional contextualisation. The implementation of the lifestyle toolkit is carried out through community health workers and an m-health platform.

#### Shraddha toolkit development and validation

- A rigorous, evidence-based process is undertaken for the development and validation of the Shraddha lifestyle toolkit.
- Multidisciplinary expert input: Inputs are gathered from experts in various fields, including medical professionals, nutritionists, physical activity specialists, mental health professionals, and cultural experts.
- Content review: The content undergoes comprehensive iterations for scientific accuracy, cultural appropriateness, and practical relevance.
- A pilot study is conducted with a small sample of individuals (*n* = 50), diagnosed with cardiometabolic multimorbidity (CMM) from the target region.
- Participant feedback: Participants are granted access to the toolkit, and their feedback is systematically collected through surveys, interviews, and focus group discussions.
- Iterative refinement: An iterative feedback loop allows for continuous fine-tuning and refinement of the toolkit based on participant input.
- Implementation through m-health platform: A digital health platform is established, featuring a two-way communication channel between participants and the study team. Health advice on different components of the toolkit is delivered through a mobile application available on the digital platform. The digital platform disseminates advice on diet and nutrition, tobacco and alcohol cessation, physical activity, medication adherence, and yoga.

#### Patient and public involvement

The co-designing of the toolkit would be through an iterative process involving qualitative interviews and Plan-Do-Study-Act cycles using the Health Belief Model for behavioural change (Table 1).

**Table 1:** Qualitative framework for formative phase

Participants	Number of FGDs/IDIs	Themes
Individuals with CMM, both male and female in various age groups	Focus group discussion with individuals in different age groups and gender 2 FGDs—males in the age group 18–60 years 2 FGDs—females in the age group 18–60 years 2 FGDs—males in the age group 60+ years 2 FGD—females in the age group 60+ years Each group will have 8–10 participants	Perception about CMM self-management, medication adherence, and control of hypertension Acceptability of a lifestyle toolkit led to control CMM
Community health workers namely accredited social health activists (ASHAs)	10–12 IDIs with ASHA workers in selected clusters	Barriers and facilitators for control of CMM Perceptions on a lifestyle toolkit to control CMM Opinions on various components to be included in the intervention
Nurses and primary care physicians	10–12 IDIs with nurses and doctors each in selected clusters	Barriers and facilitators for CMM control Perceptions on a lifestyle toolkit to control CMM Feasibility of a lifestyle toolkit intervention to control CMM and the various components that can be included

The Health Belief Model (HBM) serves as the conceptual framework for our study. HBM provides a structured understanding of how patient beliefs and perceptions influence their health behaviours and how the intervention can modify these beliefs to promote better health outcomes. The Shraddha lifestyle intervention is grounded in six core HBM constructs:

1. Perceived susceptibility: Patients of CMM need to understand that they are at a higher risk of recurrence of adverse events such as myocardial infarction, heart failure, or stroke. The Shraddha website will deliver educational broadcasts that highlight the risks associated with poor control of metabolic parameters fostering a sense of urgency for lifestyle changes and adherence to prescribed medications.
2. Perceived severity: If patients believe that the consequences of unmanaged diabetes or hypertension are severe, they may be more likely to engage in preventive behaviours. The Shraddha website will provide videos that illustrate the potential sequences of uncontrolled blood sugar and BP, such as renal failure, vision loss, or cardiovascular events, making the risks more tangible and relatable.
3. Perceived benefits: Success stories and real-life examples of reduced HbA1c levels, BP, and BMI will be shared with participants to reinforce the tangible benefits of their actions. Participants will be able to track improvements in their health indicators (e.g. HbA1c levels, BP, BMI), reinforcing the perceived benefits of the intervention.
4. Perceived barriers: Barriers may include lack of time, difficulty in accessing healthy foods, unavailability of walkways, paths, or fear of side effects from medications. The Shraddha website will provide practical, culturally tailored solutions such as meal plans and simple physical activity suggestions that can be done at home. ASHA workers will offer support during periodic home visits to address any concerns.
5. Cues to action: Reminders and alarms will serve as cues to action, prompting participants to take medications, engage in physical activity, maintain a balanced diet, and avoid tobacco and alcohol.
6. Self-efficacy: The Shraddha website will include features that allow patients to set achievable short-term goals (e.g. taking a 10-min walk each day or avoiding sugar in one meal), track their progress, and receive rewards or recognition for their efforts.

### Eligibility criteria for sites and individuals delivering interventions {10}

The start-line survey assessment would be done by accredited social health activists (ASHAs) at the household, while the Shraddha lifestyle toolkit intervention would be through the sub-centres called JAK (Janakeeya Arogya Kendram), in Kerala. Each of the sub-centres is considered a cluster for randomisation into intervention or control arm. The intervention would be carried out by staff of the JAK team consisting of a JHI, JPHN, and MLSP.

The intervention would be facilitated by a digital health platform with a two-way communication channel between participants and the study team. The developed app (Shraddha) would have modules on the different components of the toolkit accessible on handheld devices of participants. The digital platform will disseminate advice on diet and nutrition, tobacco and alcohol cessation, physical activity, medication adherence, and yoga. The development and validation of the lifestyle toolkit involve a meticulous and evidence-based process. The development phase incorporates inputs from multidisciplinary experts, including medical professionals, nutritionists, physical activity specialists, mental health professionals, and cultural experts. Firstly, experts in the relevant fields reviewed and assessed the content for scientific accuracy, cultural appropriateness, and practical relevance. Secondly, a pilot study will be conducted with a small sample of individuals with CMM from the target regions. The participants will be provided access to the toolkit, and their feedback will be systematically collected through surveys, interviews, and focus group discussions. This iterative feedback loop allowed for fine-tuning and refinement of the toolkit.

#### Components of the toolkit

- Diet and nutrition: The lifestyle toolkit includes material that educates individuals about what constitutes a healthy and nutritious diet. It provides information on the foods that should be included in their diet, such as fruits, vegetables, whole grains, lean proteins, and healthy fats. Additionally, the toolkit advises individuals on foods that should be avoided, such as processed and high-sugar foods. Emphasis is placed on consuming a balanced diet that meets their nutritional needs.
- Physical activity: The lifestyle toolkit emphasises the importance of regular physical activity for individuals with CMM. It encourages participants to exercise at least 150 min each week. This exercise can take various forms, including brisk walking, jogging, stretching exercises, or any other form of physical activity that suits their preferences and health conditions.
- Stress management: The lifestyle toolkit includes guidance on stress management through yoga practice. Yoga is recommended to reduce stress, improve mental well-being, and enhance overall health. Participants are encouraged to incorporate yoga into their daily routine to cope with stress and promote relaxation.
- Tobacco and alcohol cessation: For individuals who use tobacco or alcohol, the lifestyle toolkit offers cessation courses to help them quit these harmful habits. The toolkit provides information on the adverse effects of tobacco and alcohol consumption on cardiovascular health and encourages participants to seek support and resources to quit these habits.
- Adherence to medications: Ensuring adherence to prescribed medications is critical for managing CMM effectively. The lifestyle toolkit provides support and strategies to help individuals stay on track with their medications. This will be achieved through leveraging community health workers and a digital m-health platform, which will offer reminders, educational materials, and personalised guidance to ensure participants take their medications as prescribed.

### Outcomes {12}

#### Primary outcomes

The implementation outcomes would be studied next using the evaluation framework developed by Procter et al. for implementation outcomes that will be applied including acceptability, adoption (uptake), appropriateness, costs, feasibility, fidelity, penetration (integration of a practice within a specific setting), and sustainability.

For the digital m-health platform, our intervention will be monitored by the non-adoption, abandonment, scale-up, spread, and sustainability (NASSS) framework that is applicable across a range of technological innovations in health and social care. The proposal helps implementation of WHO-PEN protocol for self-care guidelines including utilising frontline health workers in improving self-care in patients of CMM, counselling to improving adherence and self-care, and considering patients’ beliefs and concerns about drugs and their effect.

Details of the other patient-reported outcome measures are given in Table 2.

**Table 2:** Outcome measurement tools

Objective	Variable/Outcome	Measurement tool
Acceptability/Adoption/appropriateness	Qualitative measures- Changes in perception at the level of stakeholders, participants, frontline health workers, LSG leaders Increased footfalls in the public health system (PHC, CHCs) Increased demand for drugs targeting CMM at the PHCs, CHCs, Taluk hospital of the study area	FGD Guide, In Depth Interview Guide, Pre-post audit of demand for drugs for hypertension and number of individuals following up with the public health system
Penetration	Inclusion of improving adherence to the state NCD program charter	
Fidelity	Whether participants were following intended toolkit as per instructions	Check lists, Meeting reports
Cost effectiveness	Incremental cost for an improvement in HbA1c by 0.5%	Cost of implementation
Effectiveness	Effectiveness of the lifestyle toolkit in control of disease parameters	FFQ, WHO ASSIST, MMAS-8, PSS-10, EQ- 5D -3L, BP, HbA1c, BMI measurements

#### Measurements

Blood sugar and HbA1c would be assessed with self-calibrated point-of-care (POC) devices, and weight and height with digital scales and stadiometers. The above would be measured at baseline and endline and one time-point of 6 months in between using standard procedure. Details of the other patient-reported outcome measures are given in Table 2.

### Data collection methods {18a}

Data will be collected at household (demographic data, data collected using standardised tools/questionnaires), by ASHA workers, and will be entered into a tablet PC. Before starting data collection, all research staff will be trained in the protection of human subjects, good clinical practice guidelines and administration, data entry, and analysis of study-specific data collection tools.

### Plans to promote participant retention and complete follow-up, including list of any outcome data to be collected for participants who discontinue or deviate from intervention protocols {18b}

All efforts will be made to retain all participants in the study. As they are also part of the earlier community awareness and intervention study, there is a good rapport with the study group, local self-government, and frontline health workers.

### Data management {19}

All the data collectors and research team members associated with field implementation will receive refresher training at regular intervals to ensure data quality and adherence to the intervention protocol. Monthly review and facilitation meetings will be organised at the village level to retain the participants in the intervention arm. Written informed consent will be sought from all participants. A thumb impression and signature of the witness will be taken for illiterate participants.

### Data monitoring committee {21a}

The data would be managed by the PI and the statisticians at the study centre.

### Frequency and procedures for monitoring trial conduct. If there is no monitoring, give explanation

Monthly review and facilitation meetings at ward level, refresher training, monthly quality assurance checks; the study team will oversee field implementation.

### Explanation of any interim analyses and stopping guidelines, including who will have access to these interim results and make the final decision to terminate the trial {21b}

Being a behavioural intervention, it will take 1 year for intervention, so an interim analysis is not being considered.

### Auditing {23}

Not applicable. No independent auditing is planned due to the low-risk, non-pharmacological nature of the intervention.

#### Study duration and follow-up

The study will be of 24 months including 12 months of intervention, and outcomes will be measured at baseline 6 and 12 months for primary and secondary outcomes.

### Explanation for choice of comparator {6b}

#### Study implementation

**Fig. 1:**
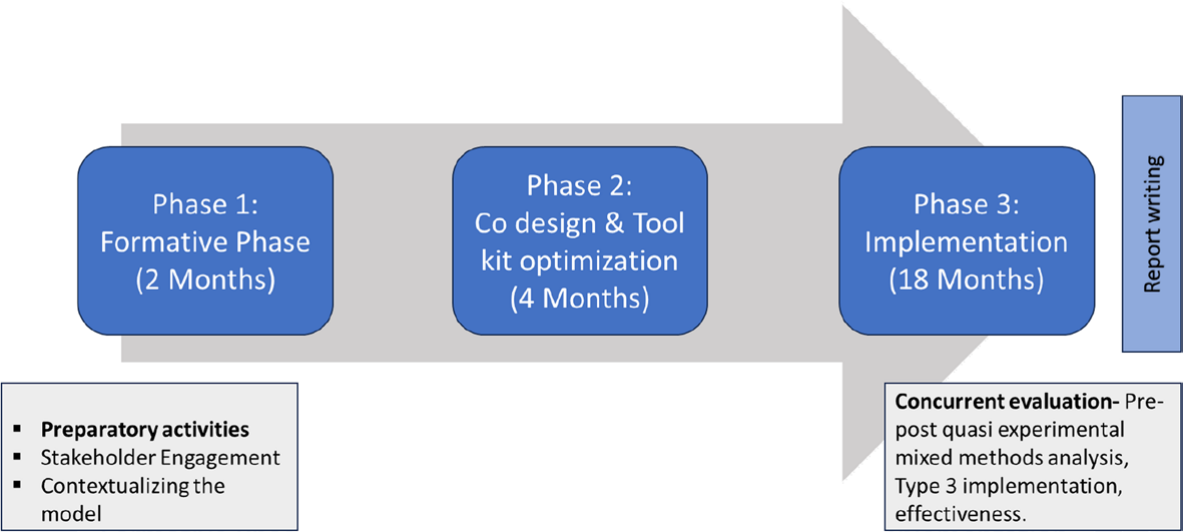
Study plan

Each sub-centre (JAK) serves as a cluster and is randomised to either the intervention (lifestyle toolkit) delivered by JAK staff (JHI, MLSP, JPHN), leveraging a digital health platform (arm A) or control arm standard of care (SoC) (arm B), by listing all the sub-centres and then using random number table for randomisation to nine clusters per arm. Each individual identified with CMM in the cluster is then consented to the study (Fig. 1).

**Fig. 2:**
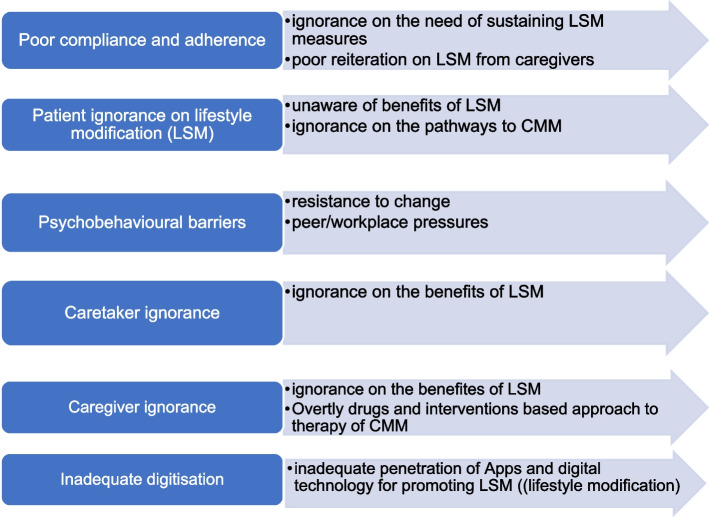
Health system challenges and gaps identified

**Fig. 3:**
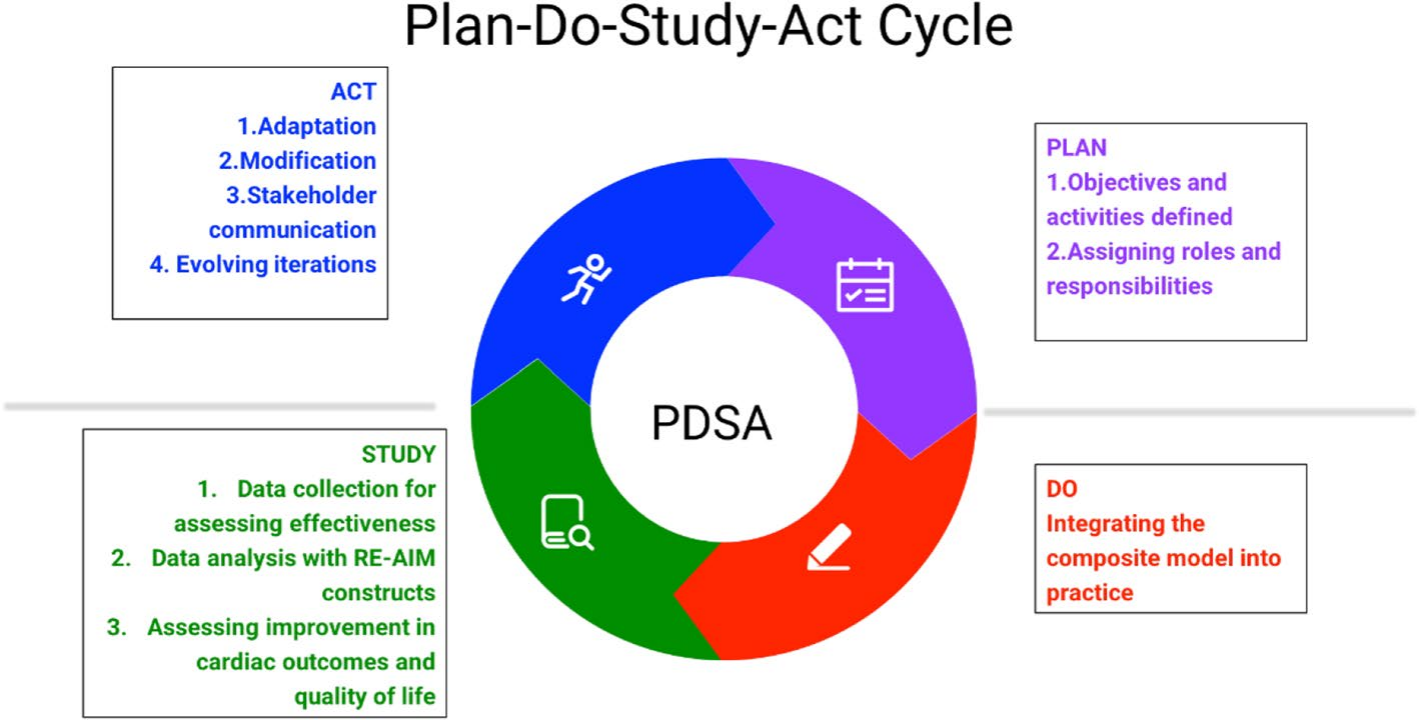
Plan-Do-Study-Act cycle

**Fig. 4:**
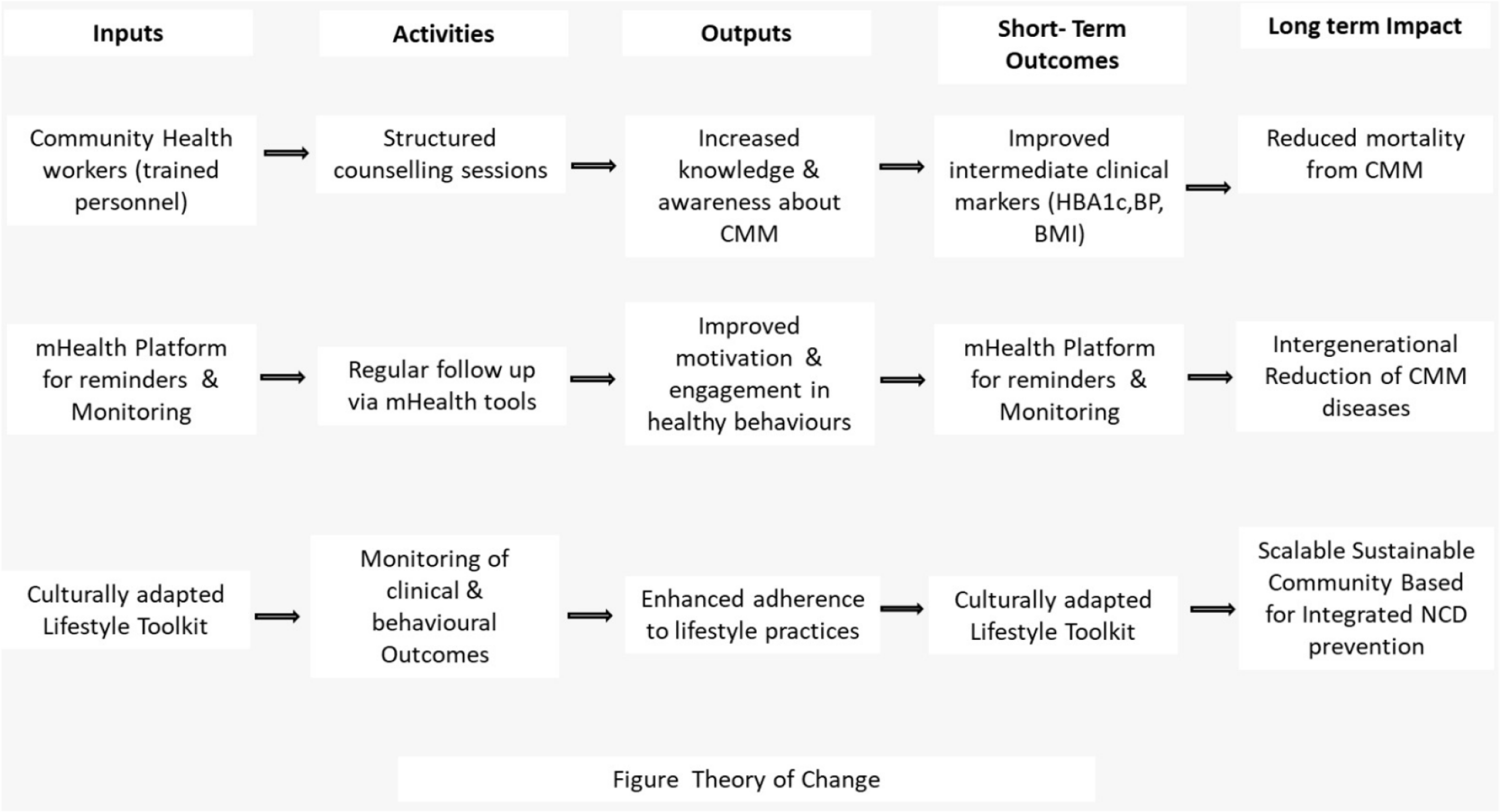
Theory of Change. Study design: mixed-methods, type III effectiveness–implementation research

**Fig. 5:**
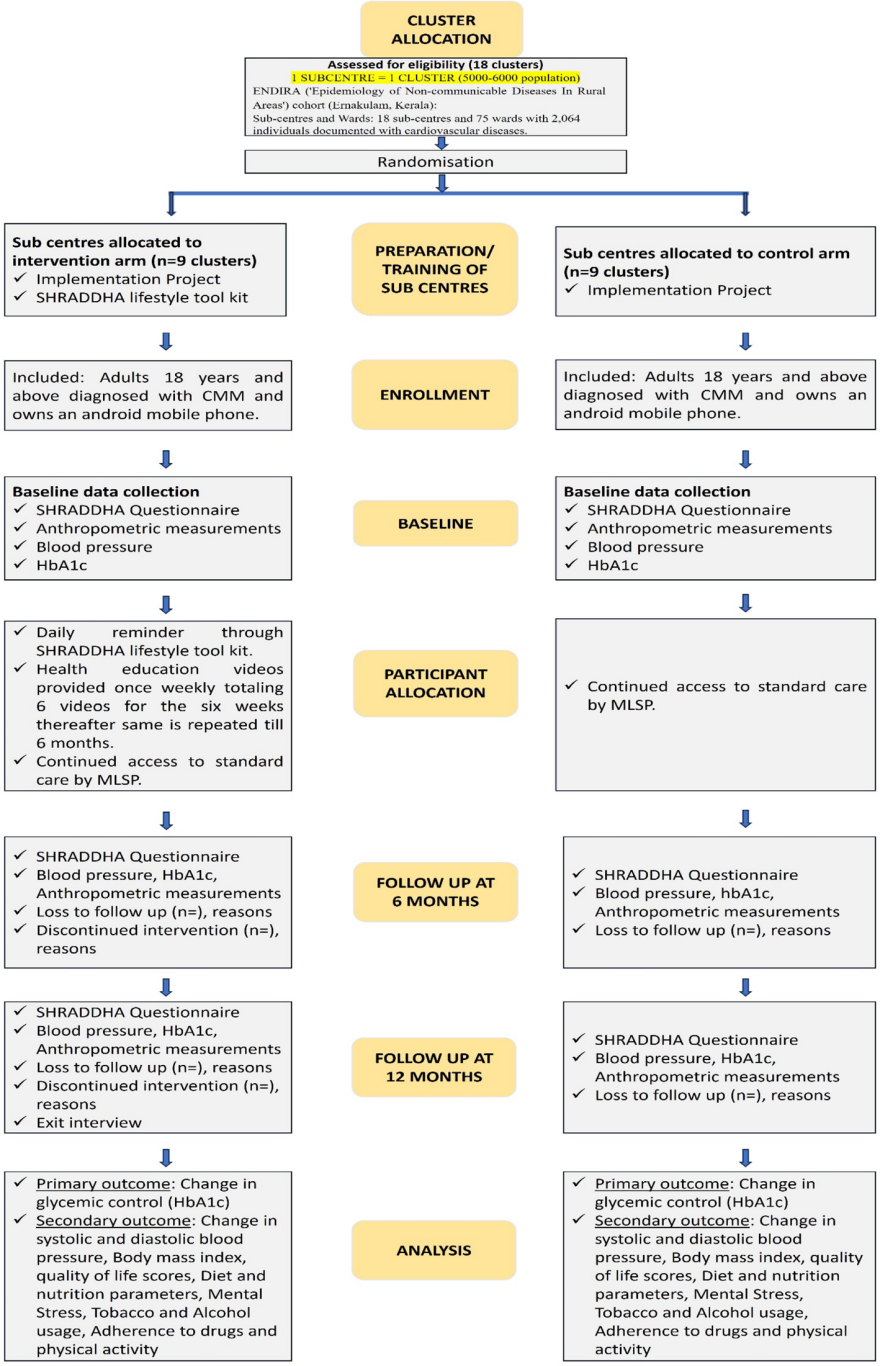
Flow chart for implementing a lifestyle toolkit versus standard of care in individuals with cardiometabolic multimorbidity

**Fig. 6:**
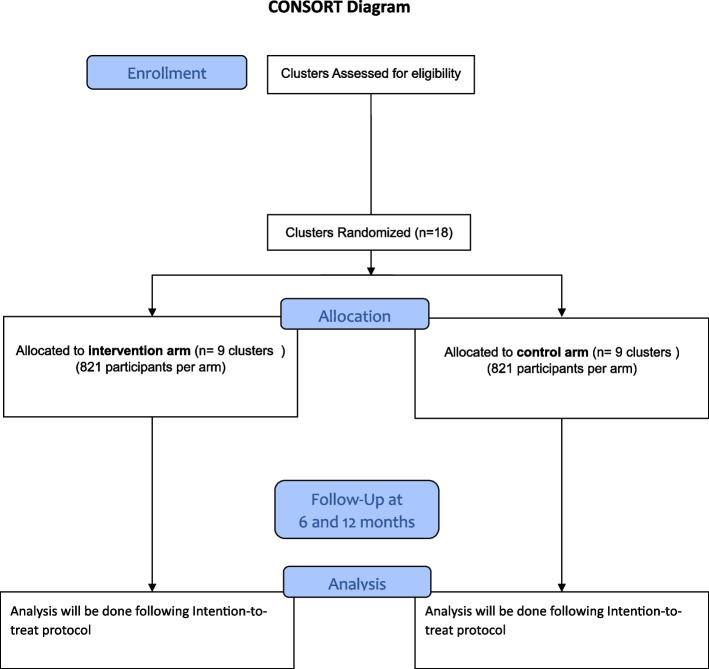
Participant flow diagram

Additionally, one first-degree relative of the index participant with CMM will be enrolled for the intergenerational preventive aspect of the proposal into the same arm of study A or B. The first-degree relative could be a male or female, ≥30 years of age, not necessarily staying in the same household, and could also be a child or sibling of the index participant (Figs. 2, 3, 4, 5, and 6).

### Criteria for discontinuing or modifying allocated interventions

Patient disinclination or dropout would be the criteria for discontinuing or modifying allocated interventions.

### Strategies to improve adherence to intervention/comparator protocols

For diet and nutrition, a Food Frequency Questionnaire would be administered at 0, 6, and 12 months. Additionally, participants would be requested to send a photograph of their food plate for calorie assessment (using the CalAI App) on three different occasions during the study period.

For physical activity, participants are encouraged to maintain log books of physical activity in minutes per day, and if available to use a step-counter to measure the number of steps per day.

For stress management, yoga sessions would be conducted online in the JAKs. A yoga trainer would schedule yoga sessions in one of the JAK centres with participants for that centre being guided in-person, while the other eight JAK centres join online for the session with participants encouraged to join from individual JAK centres. Nine such sessions of breathing exercises, postures, and mild stretching exercises are planned, one in each of the JAK centres, for the 9 weeks. The digital platform for the same has been created to facilitate the process.

For tobacco and alcohol cessation, counselling would be based on the WHO-ASSIST scores and planned accordingly with expert opinion sought in those needing the same.

For improving adherence to medications, a mix of digital prompts, tunes and jingles at weekly intervals for 4 weeks, and monthly for the next 9 months is planned as reminders to adherence and on the benefits of the same.

The intervention as envisaged is anchored in the public health system through the JAKs, with the interventions to be carried out by the JAK staff enabled and facilitated in all aspects by digital technology and a m-health platform.

In addition, each of the participants from the intervention arm has a password-encrypted access to the Shraddha app and portal which gives them access to health modules on lifestyle modifications. Participants are also given the liberty to upload health details like laboratory tests (sugar, glycated haemoglobin, etc.), or reach out with specific queries which would be addressed online in 24 h. The two-way app, to our mind, ensures ownership, participation, and would help curb dropouts.

Our previous experiences in the cohort using frontline health workers and digital platforms in the same cohort encourage us to utilise the same [34–36].

The endline survey would be done by ASHA workers, at the household, by the end of 12 months into enrolment into the study.

### Concomitant care that is permitted or prohibited during the trial

Standard concomitant care continues as directed by treating physician.

### Statistical analysis {20a}

Data will be anonymised and entered electronically on a secure storage system. Outcome data will be reported in accordance with Consolidated Standards of Reporting Trials (CONSORT) guidelines.

Multilevel modelling for repeated measurements will be used to compare the mean blood pressure (systolic and diastolic) and glycaemic levels over the study period in each site adjusted for individual covariates, where study participants are selected according to the intention-to-treat principle. Similarly, the quality of life will be assessed over and after the intervention.

A mixed-methods research approach will be used to assess the implementation according to eight domains according to the framework by Procter et al. for implementation research [37].

Quantitative measures of the implementation will be assessed by panel data regression to identify contextual factors that influence the implementation success, to identify changes throughout the study period, to identify variations and disparities in the implementation success across sites, and to evaluate the overall success of the implementation. Before the start of the study, the investigators will establish benchmarks and targets for each quantitative measure based on historical data, expert opinion, evidence-based guidelines, and stakeholders’ objectives. These targets will be used to facilitate monitoring of the implementation process and outcomes, allowing for timely adjustments and feedback.

Qualitative measures of the implementation will be assessed using constructivist grounded theory to understand how the intervention is perceived and experienced by the stakeholders and the intended beneficiaries. We will use purposive sampling to select initial participants for interviews and focus group discussions, and theoretical sampling to select additional participants and gather more data to develop and refine the emerging understanding through focused coding, memo writing, theoretical integration, reflexivity, and interpretation. We will validate the findings by engaging with participants to ensure that the theoretical framework accurately reflects their experiences and perspectives. In this way, we will identify key factors that contribute to or hinder the success of the toolkit’s implementation.

An economic evaluation of the intervention compared to SOC will consist of two analyses: (1) a cost-consequence analysis based on the observed results within the trial period and (2) a return on investment (ROI) analysis, expressing benefits in monetary terms relative to investments made, and expressing both the costs and the full range of benefits of an intervention in the same units (money), will also be conducted.

### Definition of who will be included in each analysis (e.g. all randomised participants), and in which group

Each individual with cardiometabolic multimorbidity who meets the inclusion criteria will be analysed according to the allocation of their cluster on an intention-to-treat basis.

### Methods for any additional analyses {20b}

Multilevel modelling and panel data regression to explore contextual factors; qualitative constructivist grounded theory for implementation data; economic evaluations: cost-consequence analysis and ROI analysis.

### How missing data will be handled in the analysis {20c}

Nonadherence will be managed by the intention-to-treat analysis and if there are too many missing data, imputations will be considered. Mixed method analysis will be considered for intention-to-treat analysis. Also depending on the percentage of data missingness and assumption for data missing in the study variables, appropriate missing data imputation technique will be used.

#### Study duration and follow-up

The study will be of 24 months including 12 months of intervention, and outcomes will be measured at baseline 6 and 12 months for primary and secondary outcomes.

### Harms: definition and assessment {22}

As this study is implementation of a lifestyle toolkit, no adverse events or serious adverse events and harms from the intervention are anticipated. But if there are any, they will be reported to relevant regulatory bodies such as Project Management Group, Trial Steering Committee, District Health Authority, and Ethics Committee. Trial deviations will be reported to the ethical committee.

### Ethical consideration {24}

Ethics approval for the project was granted by the IEC of AIMS-Kochi, dated ECASM-AIMS-2024-001. The clinical trial has been registered with the CTRI (Clinical Trial Registry of India) with a trial registration number of CTRI/2025/08/092529. Confidentiality of the participants will be ensured during recruitment of the participants, data collection, transcription, data analysis, and dissemination of research results. It will be done by assigning the numbers to the participants while recruiting and coding them during analysis. The data will be stored for 5 years. The results generated from this research will be freely shared with the public and research community through research publications.

### Plans for communicating important protocol modifications to relevant parties {25}

In case of protocol modifications, steps will be taken to obtain IEC approval for amendments, update CTRI, inform sponsor/funder, investigators, trial staff, and where relevant participants.

### Who will obtain informed consent or assent from potential trial participants or authorised proxies, and how {26a}

Informed consent will be taken by the accredited social health activist (ASHA) of the area who will be collecting the data. The data collection will be through an application called SHRADDHA installed in tablet PCs provided to them. The participant’s digital signature will be obtained on the tablet.

### Additional consent provisions for collection and use of participant data and biological specimens in ancillary studies, if applicable {26b}

Blood samples will be collected to assess random blood sugar and HbA1c among cardiac patients with type 2 DM after obtaining consent. These samples will be tested using point-of-care devices and will not be stored. We will request consent for review of participants’ medical records and for the collection of blood samples to assess random blood sugar and HbA1c among the cardiac patients with type 2 diabetes. But this trial does not involve collecting biological specimens for storage.

### Confidentiality {27}

Each study participant will receive a 5-digit alphanumeric unique study ID. From the app, deidentified anonymised data will be stored in Excel. This will be stored confidentially before, during, and after the trial.

### Provisions, if any, for ancillary and post-trial care, and for compensation to those who suffer harm from trial participation {30}

This is a non-pharmacological intervention; therefore, there are no specific post-trial care provisions.

## Discussion

Existing research on cardiometabolic multimorbidity (CMM) has largely been disease-centric, leaving gaps in understanding the comprehensive landscape of multi-condition management. The Shraddha lifestyle toolkit addresses this gap by providing a culturally tailored, multi-component lifestyle intervention for individuals with CMM. Its distinctiveness lies in evaluating the implementation of the *combined* effect of diet, physical activity, stress management, tobacco and alcohol cessation, and medication adherence on clinical, behavioural, and intergenerational outcomes through a cluster randomised controlled trial.

Despite the growing burden of CMM in India, research remains limited, mostly relying on secondary data [25, 27]. Global studies have linked CMM to adverse outcomes, including impaired quality of life, increased mortality, and functional decline [26, 28–31]. Prevalence estimates vary widely: 33.1% among women ≥45 years in China [38]; 8.9% in Brazil, with high rates of diabetes, stroke, and myocardial infarction [39]; and 3.5% among Canadians ≥50 years, with strong associations to inactivity and stress [29]. The intergenerational dimension is highlighted in longitudinal studies, demonstrating how early-life exposures and parental health shape offspring cardiovascular trajectories [40].

The Theory of Change (ToC) underpins the design of this protocol. The intervention assumes that structured, culturally adapted lifestyle counselling, delivered by community health workers and reinforced via mHealth support, will increase awareness, motivation, and adoption of healthy behaviours. These proximal changes are expected to improve intermediate outcomes—HbA1c, blood pressure, BMI, and medication adherence—which, over time, will lead to long-term impacts including reduced morbidity and mortality, improved quality of life, and intergenerational risk reduction. Capacity-building for frontline workers ensures sustainability, while family engagement and digital reinforcement strengthen adherence and scalability.

Current evidence supports the effectiveness of individual lifestyle interventions for diabetes, hypertension, and heart disease. The logical extension to a comprehensive toolkit addresses the shared pathways of CMM and aligns with national NCD policies, WHO ‘Best Buys’ [41], and SDGs 3.4, 3.5, and 3.8 [42]. By explicitly incorporating the intergenerational dimension, the study addresses a critical gap in existing research, exploring how family-centric interventions can interrupt the cycle of CMM risk.

Furthermore, the protocol’s design integrates implementation science principles, evaluating both clinical and process outcomes (acceptability, adoption, fidelity, coverage, cost). The ToC framework enables mapping of each intervention component to expected outputs and outcomes, guiding continuous iteration and adaptation. This ensures that the intervention is not only effective in clinical terms but also feasible, scalable, and sustainable within India’s health system.

In conclusion, by linking a novel multi-component lifestyle intervention to a clear Theory of Change, this study advances both the evidence base and implementation strategy for managing CMM. It has the potential to inform scalable, culturally appropriate, and intergenerationally mindful interventions for cardiometabolic health in India and other LMICs (Table 3).

**Table 3:** Outcome measures and tools for measurements

Outcome	Outcome measure
Diet and nutrition^a^	FFQ, app-based calorie estimation
Physical activity^b^	G-PAQ
Alcohol and tobacco use^c^	WHO-ASSIST
Adherence to drugs^d^	MMAS-8
Mental stress^e^	PSS-10
Adverse events	Death, revascularisation, rehospitalisation, reinfarction
Quality of life^f^	EQ-5D-L3
Laboratory outcomes	HbA1C
Anthropometric	Weight, BMI
Service user satisfaction^g^	PSQ 18
Resource utilisation	Intervention and treatment-related costs
Acceptability/adoption/appropriateness	Qualitative measures

^a^Cade JE, Burley VJ, Warm DL, Thompson RL, Margetts BM. Food-frequency questionnaires: a review of their design, validation and utilisation. *Nutrition Research Reviews*. 2004 Jun;17(1):5–22

^b^Sember V, Meh K, Sorić M, Starc G, Rocha P, Jurak G. Validity and reliability of international physical activity questionnaires for adults across EU countries: systematic review and meta analysis. *International Journal of Environmental Research and Public Health*. 2020 Oct;17(19):7161

^c^Newcombe DA, Humeniuk RE, Ali R. Validation of the World Health Organization Alcohol, Smoking and Substance Involvement Screening Test (ASSIST): report of results from the Australian site. *Drug and Alcohol Review*. 2005 May;24(3):217–26

^d^Tan XI, Patel I, Chang J. Review of the four item Morisky medication adherence scale (MMAS-4) and eight item Morisky medication adherence scale (MMAS-8). *INNOVATIONS in Pharmacy*. 2014;5(3):5

^e^Rajendran VG, Jayalalitha S, Adalarasu K, Usha G. A review on mental stress detection using PSS method and EEG signal method. *ECS Transactions*. 2022 Apr 24;107(1):1845

^f^Nivedita CB, Srivastava BK, Eshwar S, Jain V. Comparison of quality of life among dental caries and periodontal patients using EuroQoL-5D in KLE Society’s Institute of Dental Sciences, Bangalore: a cross-sectional study. 2018

^g^Holikatti PC, Kar N, Mishra A, Shukla R, Swain SP, Kar S. A study on patient satisfaction with psychiatric services. *Indian Journal of Psychiatry*. 2012 Oct;54(4):32

## Abbreviations

ASHA: Accredited social health activist
BMI: Body mass index
BP: Blood pressure
CHC: Community health centre
CHD: Coronary heart disease
CHW: Community health worker
CMM: Cardiometabolic multimorbidity
CONSORT: Consolidated Standards of Reporting Trials
CT: Computed tomography
CTRI: Clinical Trials Registry of India
CVD: Cardiovascular disease
DM: Diabetes mellitus
ENDIRA: Epidemiology of Non-Communicable Diseases In Rural Areas
EQ-5D: EuroQol 5-Dimension Questionnaire
FFQ: Food Frequency Questionnaire
FGD: Focus group discussion
FHC: Family health centre
HBM: Health Belief Model
HbA1c: Glycated haemoglobin
HT: Hypertension
IEC: Institutional Ethics Committee
JAK: Janakeeya Arogya Kendram
JHI: Junior health inspector
JPHN: Junior public health nurse
LSG: Local Self Government
MLSP: Mid-level service provider
MMAS: Morisky Medication Adherence Scale
NCD: Non-communicable disease
PDSA: Plan-Do-Study-Act
POC: Point-of-care
PSS: Perceived Stress Scale
RCT: Randomised controlled trial
ROI: Return on investment
SOC: Standard of care
SPIRIT: Standard Protocol Items: Recommendations for Interventional Trials
ToC: Theory of Change
WHO: World Health Organization
WHO-ASSIST: World Health Organization Alcohol, Smoking and Substance Involvement Screening Test
WHO-HEARTS: World Health Organization Technical Package for Cardiovascular Disease Management

## References

[1] Gummidi B, Gautam V, John O, Ghosh A, Jha V. Patterns of multimorbidity among a community-based cohort in rural India. J Multimorb Comorb. 2023;13:26335565221149623. 10.1177/26335565221149623. 36644651 10.1177/26335565221149623 PMC9832245

[2] Mini GK, Thankappan KR. Pattern, correlates and implications of non-communicable disease multimorbidity among older adults in selected Indian states: a cross-sectional study. BMJ Open. 2017. 10.1136/bmjopen-2016-013529. 28274966 10.1136/bmjopen-2016-013529 PMC5353268

[3] Dandona L, Dandona R, Kumar GA, Shukla DK, Paul VK, Balakrishnan K, et al. .

[4] Pati S, Swain S, Knottnerus JA, Metsemakers JFM, van den Akker M. Health related quality of life in multimorbidity: a primary-care based study from Odisha, India. Health Qual Life Outcomes. 2019;17(1):116. 10.1186/s12955-019-1180-3. 31277648 10.1186/s12955-019-1180-3 PMC6612103

[5] C R, Jeemon P. Prevalence and patterns of multi-morbidity in the productive age group of 30-69 years: a cross-sectional study in Pathanamthitta District, Kerala. Wellcome Open Res. 2020;5:233. 10.12688/wellcomeopenres.16326.1. 33215050 10.12688/wellcomeopenres.16326.1 PMC7658726

[6] Otieno P, Asiki G, Aheto JMK, Wilunda C, Sanya RE, Wami W, et al. Cardiometabolic multimorbidity associated with moderate and severe disabilities: results from the Study on Global AGEing and Adult Health (SAGE) Wave 2 in Ghana and South Africa. Glob Heart. 2023;18(1):9. 10.5334/gh.1188. 36874442 10.5334/gh.1188 PMC9983501

[7] Prabhakaran D, Jeemon P, Sharma M, Roth GA, Johnson C, Harikrishnan S, et al. The changing patterns of cardiovascular diseases and their risk factors in the states of India: the Global Burden of Disease Study 1990–2016. Lancet Glob Health. 2018;6(12):e1339–51. 10.1016/S2214-109X(18)30407-8. PubMed PMID: 30219317. 30219317 10.1016/S2214-109X(18)30407-8 PMC6227386

[8] Kalra A, Jose AP, Prabhakaran P, Kumar A, Agrawal A, Roy A, et al. The burgeoning cardiovascular disease epidemic in Indians – perspectives on contextual factors and potential solutions. Lancet Reg Health Southeast Asia. 2023. 10.1016/j.lansea.2023.100156. 37384064 10.1016/j.lansea.2023.100156 PMC10305862

[9] Jin Y, Liang J, Hong C, Liang R, Luo Y. Cardiometabolic multimorbidity, lifestyle behaviours, and cognitive function: a multicohort study. Lancet Healthy Longev. 2023;4(6):e265–73. 10.1016/S2666-7568(23)00054-5. PubMed PMID: 37150183. 37150183 10.1016/S2666-7568(23)00054-5

[10] Zhang H, Duan X, Rong P, Dang Y, Yan M, Zhao Y, et al. Effects of potential risk factors on the development of cardiometabolic multimorbidity and mortality among the elders in China. Front Cardiovasc Med. 2022. 10.3389/fcvm.2022.966217. 36158847 10.3389/fcvm.2022.966217 PMC9502033

[11] Wang LL, Wang Q, Hong Y, Ojo O, Jiang Q, Hou YY, et al. The effect of low-carbohydrate diet on glycemic control in patients with type 2 diabetes mellitus. Nutrients. 2018;10(6):661. 10.3390/nu10060661. (PubMed PMID: 29882884; PubMed Central PMCID: PMC6024764.). 29882884 10.3390/nu10060661 PMC6024764

[12] Challa HJ, Ameer MA, Uppaluri KR. DASH diet to stop hypertension. In: StatPearls. Treasure Island (FL): StatPearls Publishing; 2025. Available from: http://www.ncbi.nlm.nih.gov/books/NBK482514/ PubMed PMID: 29494120. Cited 2025 Sep 23.

[13] Pepe RB, Lottenberg AM, Fujiwara CTH, Beyruti M, Cintra DE, Machado RM, et al. Position statement on nutrition therapy for overweight and obesity: nutrition department of the Brazilian association for the study of obesity and metabolic syndrome (ABESO—2022). Diabetol Metab Syndr. 2023;15(1):124. 10.1186/s13098-023-01037-6. 37296485 10.1186/s13098-023-01037-6 PMC10251611

[14] Qin X, Chen C, Wang J, Cai A, Feng X, Jiang X, et al. Association of adiposity indices with cardiometabolic multimorbidity among 101,973 Chinese adults: a cross-sectional study. BMC Cardiovasc Disord. 2023;23(1):514. 10.1186/s12872-023-03543-x. 37865773 10.1186/s12872-023-03543-x PMC10590510

[15] Walicka M, Russo C, Baxter M, John I, Caci G, Polosa R. Impact of stopping smoking on metabolic parameters in diabetes mellitus: a scoping review. World J Diabetes. 2022;13(6):422–33. 10.4239/wjd.v13.i6.422. 35800409 10.4239/wjd.v13.i6.422 PMC9210544

[16] Zamani-Alavijeh F, Araban M, Koohestani HR, Karimy M. The effectiveness of stress management training on blood glucose control in patients with type 2 diabetes. Diabetol Metab Syndr. 2018;10(1):39. 10.1186/s13098-018-0342-5. 29760788 10.1186/s13098-018-0342-5 PMC5941598

[17] Sendekie AK, Netere AK, Kasahun AE, Belachew EA. Medication adherence and its impact on glycemic control in type 2 diabetes mellitus patients with comorbidity: a multicenter cross-sectional study in Northwest Ethiopia. PLoS One. 2022;17(9):e0274971. 10.1371/journal.pone.0274971. 36130160 10.1371/journal.pone.0274971 PMC9491880

[18] Geldsetzer P, Neve JWD, Mohan V, Prabhakaran D, Roy A, Tandon N, et al. Health system performance for multimorbid cardiometabolic disease in India: a population-based cross-sectional study. Global Heart. 2022. 10.5334/gh.1056. 35174048 10.5334/gh.1056 PMC8815445

[19] Geldsetzer P, Manne-Goehler J, Theilmann M, Davies JI, Awasthi A, Danaei G, et al. Geographic and sociodemographic variation of cardiovascular disease risk in India: a cross-sectional study of 797,540 adults. PLoS Med. 2018;15(6):e1002581. 10.1371/journal.pmed.1002581. 29920517 10.1371/journal.pmed.1002581 PMC6007838

[20] Sreeniwas Kumar A, Sinha N. Cardiovascular disease in India: a 360 degree overview. Med J Armed Forces India. 2020;76(1):1–3. 10.1016/j.mjafi.2019.12.005. (PubMed PMID: 32020960; PubMed Central PMCID: PMC6994761.). 32020960 10.1016/j.mjafi.2019.12.005 PMC6994761

[21] World Health Organisation. HEARTS: technical package for cardiovascular disease management in primary health care: risk-based CVD management. 2020. Available from: https://www.who.int/publications/i/item/9789240001367. Cited 2025 Sep 23.

[22] Dash P, Mansingh A, Nayak SR, Sahoo D, Bhattacharya D, Kanungo S, et al. Infection, cases due to SARS-CoV-2 in rural areas during early COVID-19 vaccination: findings from serosurvey study in a rural cohort of Eastern India. Epidemiol Infect. 2022;150:e58. 10.1017/S0950268822000346. (PubMed PMID: 35287778; PubMed Central PMCID: PMC8937583.). 35287778 10.1017/S0950268822000346 PMC8937583

[23] Menon J, Vijayakumar N, Joseph JK, David PC, Menon MN, Mukundan S, et al. Below the poverty line and non-communicable diseases in Kerala: the Epidemiology of Non-communicable Diseases in Rural Areas (ENDIRA) study. Int J Cardiol. 2015;187:519–24. 10.1016/j.ijcard.2015.04.009. 25846664 10.1016/j.ijcard.2015.04.009

[24] Menon J, Joseph J, Thachil A, Attacheril TV, Banerjee A. Surveillance of noncommunicable diseases by community health workers in Kerala: the Epidemiology of Non-communicable Diseases in Rural Areas (ENDIRA) study. Global Heart. 2014;9(4):409–17. 10.1016/j.gheart.2014.07.003. 25592794 10.1016/j.gheart.2014.07.003

[25] Puri P, Singh SK, Pati S. Identifying non-communicable disease multimorbidity patterns and associated factors: a latent class analysis approach. BMJ Open. 2022;12(7):e053981. 10.1136/bmjopen-2021-053981. (PubMed PMID: 35820748; PubMed Central PMCID: PMC9277367.). 35820748 10.1136/bmjopen-2021-053981 PMC9277367

[26] Otieno P, Asiki G, Wekesah F, Wilunda C, Sanya RE, Wami W, et al. Multimorbidity of cardiometabolic diseases: a cross-sectional study of patterns, clusters and associated risk factors in Sub-Saharan Africa. BMJ Open. 2023;13(2):e064275. 10.1136/bmjopen-2022-064275. (PubMed PMID: 36759029; PubMed Central PMCID: PMC9923299.). 36759029 10.1136/bmjopen-2022-064275 PMC9923299

[27] Sinha A, Kerketta S, Ghosal S, Kanungo S, Lee JT, Pati S. Multimorbidity and complex multimorbidity in India: findings from the 2017–2018 Longitudinal Ageing Study in India (LASI). Int J Environ Res Public Health. 2022;19(15):9091. 10.3390/ijerph19159091. 35897461 10.3390/ijerph19159091 PMC9332385

[28] Joseph JJ, Rajwani A, Roper D, Zhao S, Kline D, Odei J, et al. Associations of cardiometabolic multimorbidity with all-cause and coronary heart disease mortality among Black adults in the Jackson Heart Study. JAMA Netw Open. 2022;5(10):e2238361. 10.1001/jamanetworkopen.2022.38361. 36282500 10.1001/jamanetworkopen.2022.38361 PMC9597394

[29] Sakakibara BM, Obembe AO, Eng JJ. The prevalence of cardiometabolic multimorbidity and its association with physical activity, diet, and stress in Canada: evidence from a population-based cross-sectional study. BMC Public Health. 2019;19(1):1361. 10.1186/s12889-019-7682-4. 31651286 10.1186/s12889-019-7682-4 PMC6814029

[30] Sewpaul R, Mbewu AD, Fagbamigbe AF, Kandala NB, Reddy SP. Prevalence of multimorbidity of cardiometabolic conditions and associated risk factors in a population-based sample of South Africans: a cross-sectional study. Public Health Pract. 2021;30(2):100193. 10.1016/j.puhip.2021.100193. 10.1016/j.puhip.2021.100193 PMC9461539 36101622

[31] Zhao Y, Zhang H, Liu X, Desloge A, Wang Q, Zhao S, et al. The prevalence of cardiometabolic multimorbidity and its associations with health outcomes among women in China. Front Cardiovasc Med. 2023;9(10):922932. 10.3389/fcvm.2023.922932. 10.3389/fcvm.2023.922932 PMC9947472 36844741

[32] Savelieva K, Pulkki-Råback L, Jokela M, Kubzansky LD, Elovainio M, Mikkilä V, et al. Intergenerational transmission of socioeconomic position and ideal cardiovascular health: 32-year follow-up study. Health Psychol Off J Div Health Psychol Am Psychol Assoc. 2017;36(3):270–9. 10.1037/hea0000441. (PubMed PMID: 27929335). 10.1037/hea0000441 27929335

[33] Griffin SJ, Borch-Johnsen K, Davies MJ, Khunti K, Rutten GEHM, Sandbæk A, et al. Effect of early intensive multifactorial therapy on 5-year cardiovascular outcomes in individuals with type 2 diabetes detected by screening (ADDITION-Europe): a cluster-randomised trial. Lancet. 2011;378(9786):156–67. 10.1016/S0140-6736(11)60698-3. 21705063 10.1016/S0140-6736(11)60698-3 PMC3136726

[34] Menon J, Numpeli M, Kunjan SP, Karimbuvayilil BV, Sreedevi A, Panniyamakkal J, et al. A sustainable community-based model of noncommunicable disease risk factor surveillance (Shraddha-Jagrithi Project): protocol for a cohort study. JMIR Res Protoc. 2021;10(10):e27299. 10.2196/27299. 34677141 10.2196/27299 PMC8571687

[35] Banerjee A, Menon JC, KrishnaKumar R, Ravikumar B, Srinivas R, Ladikas M. A learning health system for secondary prevention in cardiovascular disease in Kerala using informatics and non-physician health workers (LHS-CVD). Indian Heart J. 2018;1(70):S2. 10.1016/j.ihj.2018.10.007.

[36] Menon JC, John D, Sreedevi A, Janakiram C, R A, S S, et al. Improving medication adherence among persons with cardiovascular disease through m-health and community health worker-led interventions in Kerala; protocol for a type II effectiveness-implementation research-(SHRADDHA-ENDIRA). Trials. 2024;25(1):437. 10.1186/s13063-024-08244-0. PubMed PMID: 38956612; PubMed Central PMCID: PMC11221042. 10.1186/s13063-024-08244-0 PMC11221042 38956612

[37] Proctor E, Silmere H, Raghavan R, Hovmand P, Aarons G, Bunger A, et al. Outcomes for implementation research: conceptual distinctions, measurement challenges, and research agenda. Adm Policy Ment Health. 2011;38(2):65–76. 10.1007/s10488-010-0319-7. 20957426 10.1007/s10488-010-0319-7 PMC3068522

[38] Han Y, Hu Y, Yu C, Guo Y, Pei P, Yang L, et al. Lifestyle, cardiometabolic disease, and multimorbidity in a prospective Chinese study. Eur Heart J. 2021;42(34):3374–84. 10.1093/eurheartj/ehab413. 34333624 10.1093/eurheartj/ehab413 PMC8423468

[39] Batista SR, Vitorino PVO, Silva RR, Sousa ALL, Barroso WKS, Coca A. Cardiovascular multimorbidity and associated factors: the first Brazilian Registry of patients with hypertension. Eur Heart J. 2021;42(Supplement_1):ehab724.2337. 10.1093/eurheartj/ehab724.2337.

[40] Jensen TM, Duke NN, Harris KM, Hotz VJ, Perreira KM. Like parent, like child: intergenerational patterns of cardiovascular risk factors at midlife. J Adolesc Health. 2021;68(3):596–603. 10.1016/j.jadohealth.2020.06.039. 32753345 10.1016/j.jadohealth.2020.06.039 PMC7854782

[41] World Health Organisation. More ways, to save more lives, for less money: World Health Assembly adopts more Best Buys to tackle noncommunicable diseases. Available from: https://www.who.int/news/item/26-05-2023-more-ways--to-save-more-lives--for-less-money----world-health-assembly-adopts-more-best-buys--to-tackle-noncommunicable-diseases. Cited 2025 Sep 24.

[42] Martin. Health. United Nations sustainable development. Available from: https://www.un.org/sustainabledevelopment/health/. Cited 2025 Sep 24.

